# AZP2006, a new promising treatment for Alzheimer’s and related diseases

**DOI:** 10.1038/s41598-021-94708-1

**Published:** 2021-08-19

**Authors:** N. Callizot, C. Estrella, S. Burlet, A. Henriques, C. Brantis, M. Barrier, M. L. Campanari, P. Verwaerde

**Affiliations:** 1 Alzprotect, Parc Eurasanté, 85C rue Nelson Mandela, 59120 Loos, France; 2 Neuro-Sys, 410 Chemin Départemental 60, 13120 Gardanne, France

**Keywords:** Cell biology, Drug discovery, Neuroscience, Diseases, Pathogenesis

## Abstract

Progranulin (PGRN) is a protein with multiple functions including the regulation of neuroinflammation, neuronal survival, neurite and synapsis growth. Although the mechanisms of action of PGRN are currently unknown, its potential therapeutic application in treating neurodegenerative diseases is huge. Thus, strategies to increase PGRN levels in patients could provide an effective treatment. In the present study, we investigated the effects of AZP2006, a lysotropic molecule now in phase 2a clinical trial in Progressive Supranuclear Palsy patients, for its ability to increase PGRN level and promote neuroprotection. We showed for the first time the in vitro and in vivo neuroprotective effects of AZP2006 in neurons injured with Aβ_1–42_ and in two different pathological animal models of Alzheimer’s disease (AD) and aging. Thus, the chronic treatment with AZP2006 was shown to reduce the loss of central synapses and neurons but also to dramatically decrease the massive neuroinflammation associated with the animal pathology. A deeper investigation showed that the beneficial effects of AZP2006 were associated with PGRN production. Also, AZP2006 binds to PSAP (the cofactor of PGRN) and inhibits TLR9 receptors normally responsible for proinflammation when activated. Altogether, these results showed the high potential of AZP2006 as a new putative treatment for AD and related diseases.

## Introduction

Alzheimer’s disease (AD) is the most frequent cause of dementia in elderly populations^1^. This pathology affects almost 50 million people worldwide, and the incidence will increase in the next years. The main features of the pathology are the accumulation of amyloid beta (Aβ) plaques and neurofilament tangles (aggregates of hyperphosphorylated Tau protein) associated with a loss of synapses, neurites and finally neuronal death^2^.

Recent evidence supports that soluble Aβ oligomers are the primary pathogenic drivers of neurodegeneration in AD and further proposes that soluble Aβ oligomers cooperate with pathological Tau to progressively degenerate the learning and memory circuitry required for cognitive functions^3^. In addition to neurotoxic Aβ and Tau, the other critical risk factor for clinical onset of AD is aging. In this context, age-related mitochondrial damage appears to play an important role in Aβ/Tau induced neurodegeneration in AD^4^. Aggregation of the Tau protein is also a neuropathological hallmark of many other neurodegenerative disorders classified as tauopathies, including frontotemporal dementia (FTD), corticobasal degeneration (CBD), progressive supranuclear palsy (PSP) and Pick’s disease (PiD)^5,6^. While mutations in the Tau gene (MAPT) are known to cause primary tauopathies, no MAPT mutations directly cause AD^7^. The A152T mutation is the only tau associated mutation linked to AD. This mutation is also linked with Lewy bodies dementia (DLB)^8^ and the spectrum of frontotemporal dementia disorders, including PSP and CBD^7^.

Neuroinflammation and abnormal secretion of proinflammatory cytokines also contribute to neuronal dysfunction and neuronal death, without being the initial cause of AD or related diseases (PSP, FTD)^9–11^.

Progranulin (PGRN) is a glycoprotein that can be secreted or transported to the lysosome in the immune system and neurons^12,13^. It is involved in multiple physiological processes such as neuroinflammation, neurite branching and outgrowth, lysosomal functions and neuronal survival^14^. PGRN high production and breakdown into granulin is linked to pathological conditions^15^ and its low expression (or reduced circulating levels) causes neurodegeneration such as observed in GRN frontotemporal dementia (GRN-FTD)^16–18^. Increasing PGRN levels in animal models of FTD, AD or PD have been reported to reduce both pathological and clinical features^19–21^. Therefore, strategies aiming to increase PGRN levels, and preventing its degradation into granulin peptides, are highly investigated as potential therapeutic approaches.

AZP2006 (INN: Ezeprogind) is a small molecule (N-3-(4-(3-(diisobutylamino)propyl)piperazin-1-yl)propyl)-1H-benzo[d]imidazol-2-amine di-sulphate salt) under development for PSP (currently in phase 2a in patients, ClinicalTrials.gov Identifier: NCT04008355) and under preclinical development for the treatment of AD and related disorders. AZP2006 was the lead compound of a piperazine family and was initially screened and selected for its ability to reduce the release of Aβ species and to increase the amount of APP metabolites^22,23^.

In the present study, we showed for the first time the correlation between PGRN and the AZP2006 neuroprotective effects in a in vitro model of acute amyloid-β_1–42_ (Aβ_1–42_) injuries where rat or mice’s fetus primary cortical neurons were cultured with microglia in presence of Aβ_1–42_ oligomers.

In this model, treatment with AZP2006 significantly decreased Aβ_1–42_-induced impairments (synapse, neurite and neuronal loss as well as a microglia activation) and lost its effects in absence of PGRN.

Also, we tested AZP2006 in two in vivo models relevant for AD and aging (induced with Aβ_25–35_ peptide and SAMP8 mouse model respectively). We showed that AZP2006 was able to rescue the behavioral and biochemical impairments measured in different animal ages and treatment periods. The chronic treatment with AZP2006 was shown to reduce the loss of central synapses and neurons but also to dramatically decrease the neuroinflammation associated with the animal pathology.

Altogether our findings reinforce the pharmaceutical relevance of AZP2006 and support its clinical development in the treatment of AD and other related neurodegenerative diseases.

## Results

### AZP2006 is able to prevent neuronal death induced by Aβ injury, and to reduce the associated microglial activation

In the present work we used our established model of rat cortical neurons cultured in presence of microglial cells, the primary immune effector cells of the brain, after Aβ_1–42_ intoxication^24^.

The application of Aβ is well known to induce neurite loss and neuronal cell death associated with a massive oxidative stress and apoptotic pathway activation (for details see^25^).

**Figure 1:**
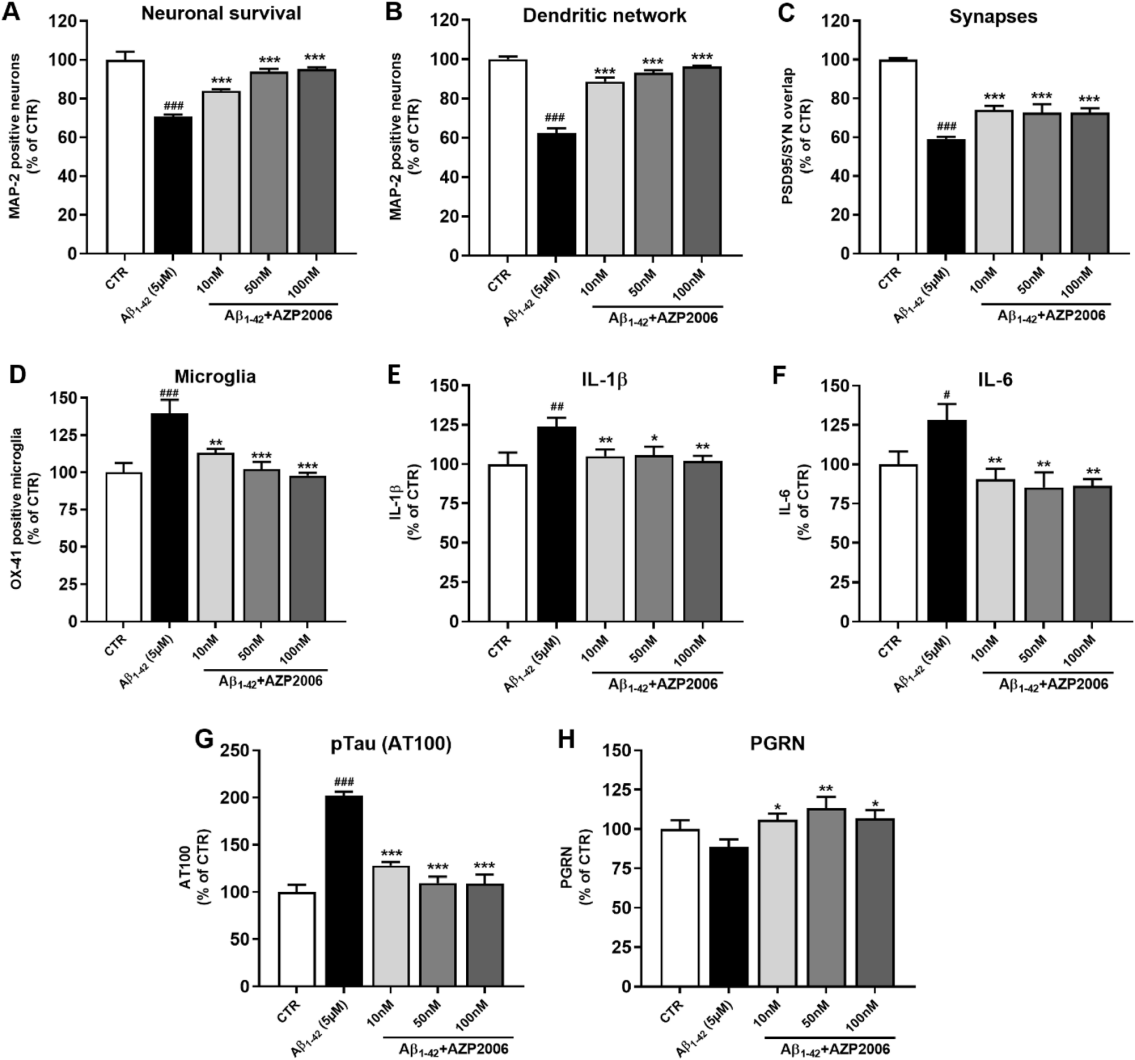
Aβ oligomers exposition induces AD pathological signatures and microglia activation which are totally rescued by AZP2006 treatment.** (A**, **B**) After 72 h of Aβ_1-42_ exposure, the number of rat cortical neurons, culture with microglia, is reduced (MAP-2 staining; CTR = 49 positive soma, ± 2, n = 6) and dendritic morphology changed to a less complex network (CTRL = 15,365 µm/well ± 200, n = 6). The presence of AZP2006 in the same medium rescue neurons survival and morphology. All values are expressed as mean ± SEM (standard error of the mean). One-way ANOVA followed by Dunnett’s test, n = 4–6. ^#^*p* < 0.05 versus CTR. *p < 0.05 versus Aβ_1-42_ condition. (**C**) Neurons were stained with postsynaptic marker PSD95 and presynaptic marker synaptophysin; their overlap was automatically quantified (overlapping PSD95/SYN, μm^2^ of overlapping) to obtain the total synapses area. *CTR* = 242 µm^2^/well, ± 1.5, n = 5. All values are expressed as mean ± SEM (standard error of the mean). One-way ANOVA followed by PLSD Fisher’s test, n = 4–5. ^#^*p* < 0.05 versus CTR. *p < 0.05 versus Aβ_1-42_ condition. (**D**) The immunostaining quantification for OX-41, an antibody clone which specifically recognizes macrophages and granulocytes, show microglia activation, reduced after AZP2006 treatment. *CTR* = 3134 µm^2^/well, ± 197.3 n = 5. All values are expressed as mean ± SEM (standard error of the mean). One-way ANOVA followed by PLSD Fisher’s test, n = 5–6. ^#^*p* < 0.05 versus CTR. *p < 0.05 versus Aβ_1-42_ condition. (**E, F**) Released IL-1β and IL-6 protein quantification (supernatant of cell culture) from rat cortical neurons and microglia exposed to Aβ_1-42_ oligomers for 72 h with or without AZP2006. *CTR IL-1β* = 0.065 pg/ml/cell, ± 0.005, n = 5 ; *CTR IL-6* = 0.10 4 pg/ml/cell, ± 0.008, n = 4. All values are expressed as mean ± SEM (standard error of the mean). One-way ANOVA followed by PLSD Fisher’s test, n = 4–6. ^#^*p* < 0.05 versus CTR. *p < 0.05 versus Aβ_1-42_ condition. (**G)** AT100 area in neurites of cortical neurons cultured with microglia, injured with _Aß1-42_ (5 µM, 72 h) and treated with AZP2006. *CTR* = 1.751 µm^2^ (mean *per* neurite/well), ± 0.13, n = 5. All values are expressed as mean ± SEM (standard error of the mean). One-way ANOVA followed by PLSD Fisher’s test, n = 4–6. ^#^*p* < 0.05 versus CTR. *p < 0.05 versus Aβ_1-42_ condition. (**H)** After 72 h of treatment, neuron lysates were removed and analyzed for PGRN protein level. *CTR* = 1.917 ng/ml, ± 0.109, n = 6. All values are expressed as mean ± SEM (standard error of the mean). One-way ANOVA followed by PLSD Fisher’s test, n = 5–6. ^#^*p* < 0.05 versus CTR. *p < 0.05 versus Aβ_1-42_ condition.

As a first step, in order to guarantee the validity of our model, we assessed the neuronal cell number and morphology by MAP-2 immunostaining. As expected, after 72 h of Aβ_1–42_ exposure, we observed a 40% of reduction in neuron survival and dendritic network respect to control neurons (Fig. 1A, B, *black column* and supplementary Fig. 1A). Then, we tested the effect of AZP2006 treatment given during Aβ_1–42_ intoxication. Surprisingly, after 72 h, AZP2006 was able to improve neuronal survival and to maintain the integrity of the dendritic network to normal level at all tested concentrations (10, 50 and 100 nM, Fig. 1A, B, *grey columns* and supplementary Fig. 1A).

To test the effect of AZP2006 on synapses we performed double immunostaining for postsynaptic protein 95 (PSD95) and synaptophysin (SYN, a presynaptic vesicle protein). These markers measured together provide a good indicator of synaptic growth and activity. Interestingly, in presence of AZP2006, the intensity of PSD95 and SYN was higher with respect to Aβ_1–42_ condition (Fig. 1C) suggesting a role for AZP2006 in synapse protection or rescue.

Compelling evidence from histological examination has demonstrated the role that Aβ plays as inducer of microglia activation and neuroinflammation^26–28^. Also in our model, Aβ_1–42_ caused the microglia activation measured by OX-41-increased staining (Fig. 1D) associated with an augmented release of pro-inflammatory cytokines interleukin 1β (IL-1β), 6 (IL-6) (Fig. 1E, F) and TNFα (supplementary Fig. 1B). Importantly, AZP2006 significantly decreased OX-41 staining, IL-1β, IL-6 and TNFα cytokine levels in the cell supernatants, suggesting its anti-inflammatory role in response to Aβ intoxication.

We also monitored Tau phosphorylation^29^ and found that AZP2006 was able to significantly decrease the Aβ_1–42_-induced AT100 immunolabeling to normal level compared with the control (Fig. 1G).

Due to the AZP2006-induced effects, we decided to measure the expression levels of brain-derived neurotrophic factor (BDNF) and PGRN, whose trophic role in neurons has been extensively explored^30,31^. In our model, both BDNF and PGRN were reduced by Aβ_1–42_. But, while AZP2006 had no ameliorative effect on BDNF (Supplementary Fig. 1C), it was able to significantly increase PGRN release (Fig. 1H and Supplementary Fig. 2A), suggesting a neuroprotective effect probably linked to the latter.

### Loss of soluble PGRN abolishes AZP2006 neuroprotective effects

To test whether the effects of AZP2006 were via PGRN-related mechanisms, we measured neuronal survival (cell number) and dendritic network morphology in mouse neurons injured with Aβ peptide and treated with AZP2006 in absence of released PGRN.

For this purpose, we added a specific antibody (ref: MAB2557) to the culture medium to sequester PGRN.

**Figure 2:**
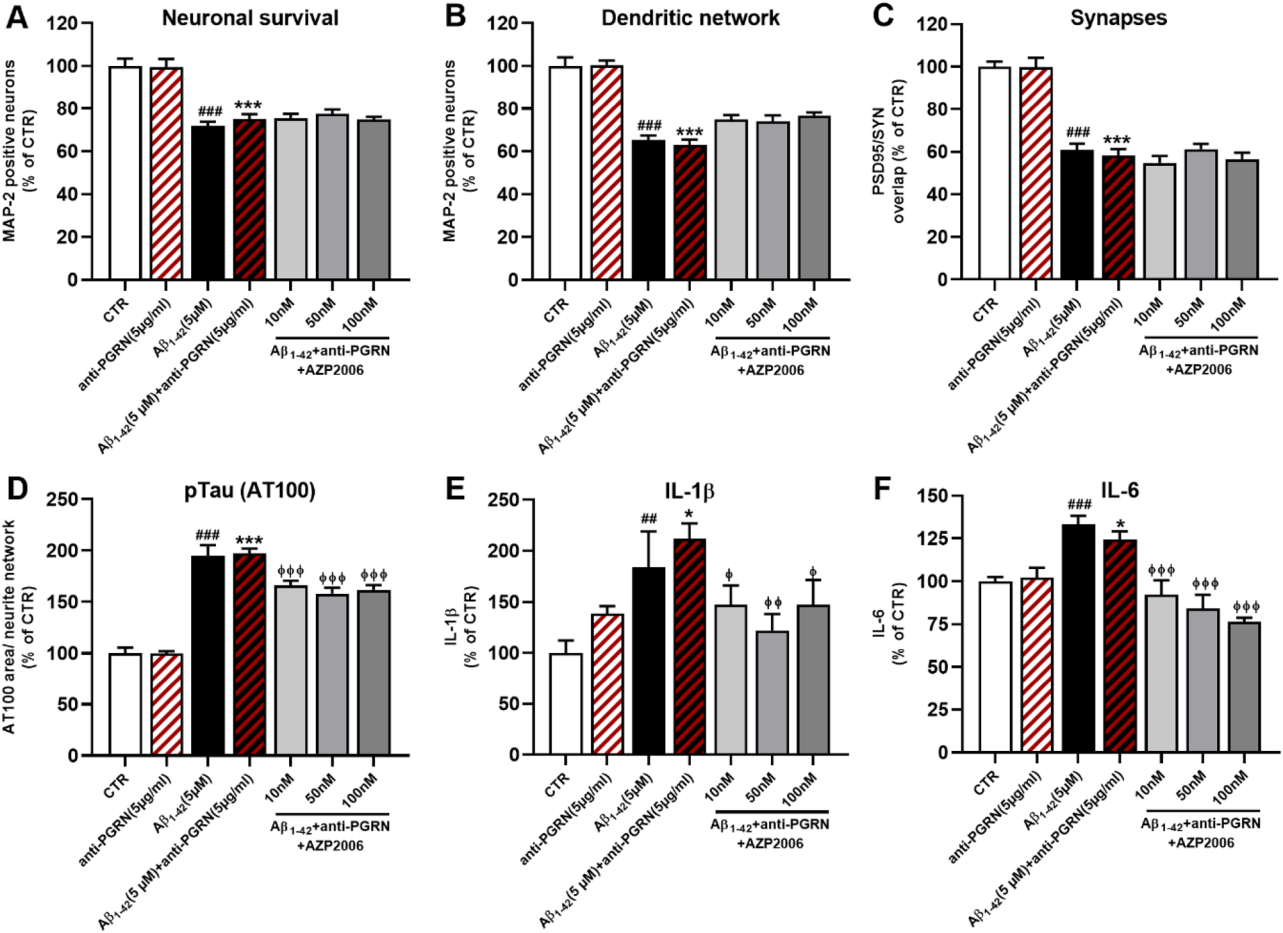
AZP2006/PGRN-induced protection on neuronal survival and morphology.** (A**, **B**) Neuronal cell number and neurite network in mouse cortical neurons intoxicated with Aβ_1-42_ oligomers (black bar) and treated with different concentrations of AZP2006 (grey bars) in absence of PGRN (antibody sequestration). MAP-2 staining; CTR (A) = 34.3 positive soma, ± 1.18, n = 6; CTRL (B) = 5883 µm/well ± 239, n = 6). (**C**) Synapses were measured by the overlap of PSD95 and synaptophysin. *CTR* = 598 µm^2^/well, ± 14.7, n = 6. (**D**) Immunohistochemical quantification of hyperphosphorylated Tau. After AZP2006 treatment, it decreases independently of PGRN sequestration. *CTR* = 0.34 µm^2^ (mean *per* neurite/well), ± 0.087, n = 6. (**E**, **F**) IL-1β and IL-6 protein quantification in the supernatant of cortical neurons exposed to Aβ_1-42_ oligomers for 72 h with or without AZP2006 and PRG antibody. *CTR IL-1β* = 0.048 pg/ml/cell, ± 0.0061, n = 5; *CTR IL-6* = 0.104 pg/ml/cell, ± 0.004, n = 5. All values are expressed as mean ± SEM (standard error of the mean). One-way ANOVA followed by PLSD Fisher’s test, n = 5–6. ^#^*p* < 0.05 versus CTR; *p < 0.05 versus anti-PGRN (5 µg/ml); ɸ *p* < 0.05 versus Aβ_1-42_ (5 µM) + anti-PGRN (5 µg/ml) condition.

In our model, extracellular PGRN loss totally abolished the neuroprotective effects of AZP2006 on neuronal survival, dendritic network and synapses integrity (Fig. 2A–C and Supplementary Fig. 2B).

By contrast, the absence of PGRN didn’t affect to the same extent the negative regulation that AZP2006 plays on Tau hyperphosphorylation (Fig. 2D), as well as on neuroinflammation activation (Fig. 2E, F) suggesting the existence of PGRN-independent mechanisms of actions.

### AZP2006 is able to bind to the PGRN/PSAP complex

**Figure 3:**
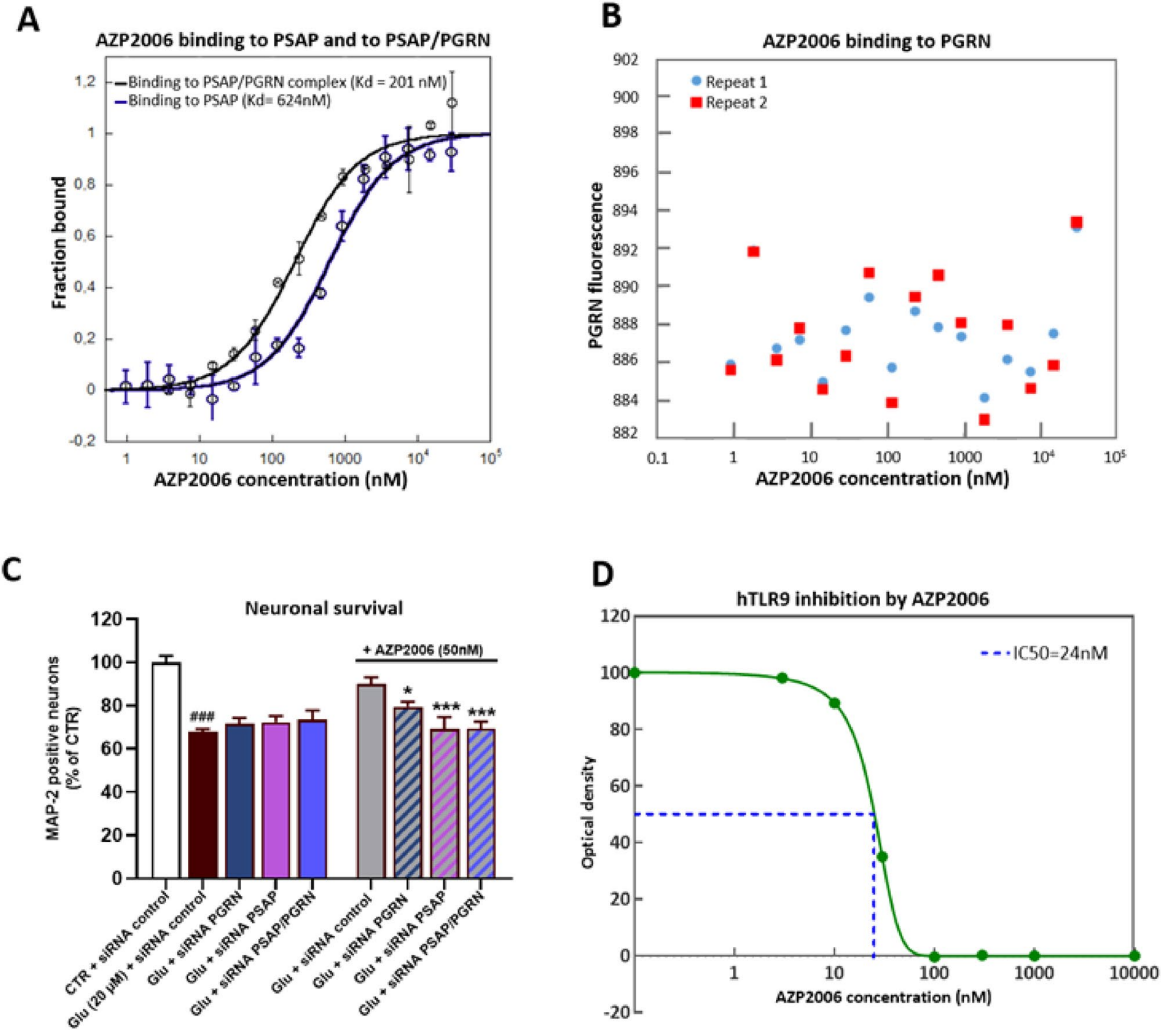
MST analysis of the interactions between AZP2006, PGRN and PSAP. (**A**, **B**) The compound AZP2006 was added in increasing concentrations to the two RED-labeled peptides (PGRN and PSAP) that served as targets. Raw fluorescence data were normalized to the fraction of bound target. Error bars represent standard deviations from two individual repeat measurements. The Kd was determined from curve fitting according to the Hill equation^77^. (**C**) The positive effect of AZP2006 on rat cortical neurons survival is totally abolished by PGRN and PSAP knockdown (24 h of siRNA transfection). CTR + siRNA control = 54.17 positive soma, ± 1.49, n = 6. All values are expressed as mean ± SEM (standard error of the mean). One-way ANOVA followed by PLSD Fisher’s test, n = 5–6. #*p* < 0.05 versus CTR + siRNA control ; *p < 0.05 versus Glu (20 µM) + siRNA control. (**D**) dose/response analysis for AZP2006 on hTLR9 activity. All tested doses of ALZ2066 (3, 10, 30, 100, 300 and 1000 nM) were able to diminish hTLR9 activity up to totally antagonize its function at 100 nM (IC50 = 24 nM).

Within the cell, PGRN localizes to the lysosomal compartment where it directly binds another lysosomal protein, the prosaposin (PSAP), essential for glycosphingolipid degradation^12,32^. Also, AZP2006 has high affinity for the lysosome vesicles (LAMP1 positive) and accumulates into these organelles (Supplementary Fig. 3A). Therefore, we tested the ability of AZP2006 to bind PGRN/PSAP complex by Fluorescent MST competition assay. As shown in Fig. 3A, AZP2006 was able to bind the PGRN/PSAP complex with a higher affinity (Kd = 201 nM) than to PSAP (Kd = 624 nM). No interaction was measured between AZP2006 and PGRN alone (Fig. 3B), suggesting the existence of interaction inside the lysosome.

Next, we used RNA interference (siRNA) to specifically induce silencing of PGRN and PSAP genes in rat cortical neurons to explore the necessity of the PGRN/PSAP complex in mediating AZP2006 protective action (Fig. 3C and Supplementary Fig. 3B). When administrated alone or with a mismatch siRNA (siRNA CT), AZP2006 protected neurons from glutamatergic stress. However, when preceded by knockdown of endogenous PGRN, PSAP or the combination of both of them (siRNA PSAP/PGRN), AZP2006 lost its effect and the number of MAP-2 positive neurons decreased to a level comparable to glutamate condition.

Finally, we explored the effect of AZP2006 on Toll-like receptor 9 (TLR9) activity. Into the lysosome in fact, PGRN can be cleaved in granulins that in turn are implicated in pro-inflammatory action through the activation of TLR9^33,34^.

For that we used a recombinant cell line (HEK-293) overexpressing the human TLR9 and the reporter gene ALP under the control of NFkB promoter. Normally NFkB promoter is induced by TLR9 activation after its direct contact with CpG oligodeoxynucleotides (ODNs).

As shown in Fig. 3D, the TLR9 activity decreased by increasing AZP2006 concentrations even in presence of ODNs. These results suggested a possible interaction between AZP2006 and TLR9 which leads to TLR9 inactivation and the interleukins decrease previously seen in absence of PGRN.

### AZP2006 treatment is able to prevent Aβ_25–35_ damages in AD mice model

The efficacy of AZP2006 treatment was evaluated in vivo in an Alzheimer’s disease model, where mice were injured with intracerebral injection of Aβ_25–35_ peptide as previously described^35,36^. AZP2006 was given orally by daily gavage and improvement was measured at the spatial working memory (spontaneous alternation in the Y-maze) and in spatial learning and memory (NOR test) (see design in Supplementary Fig. 4A).

Aβ_25–35_ injection and/or treatment with AZP2006 from D0, D01 or D04 showed no effect on speed, anxiety, or stereotypic behavior in the open-field procedure in mice measured during session 1 at D08 (Supplementary Fig. 5).

**Figure 4:**
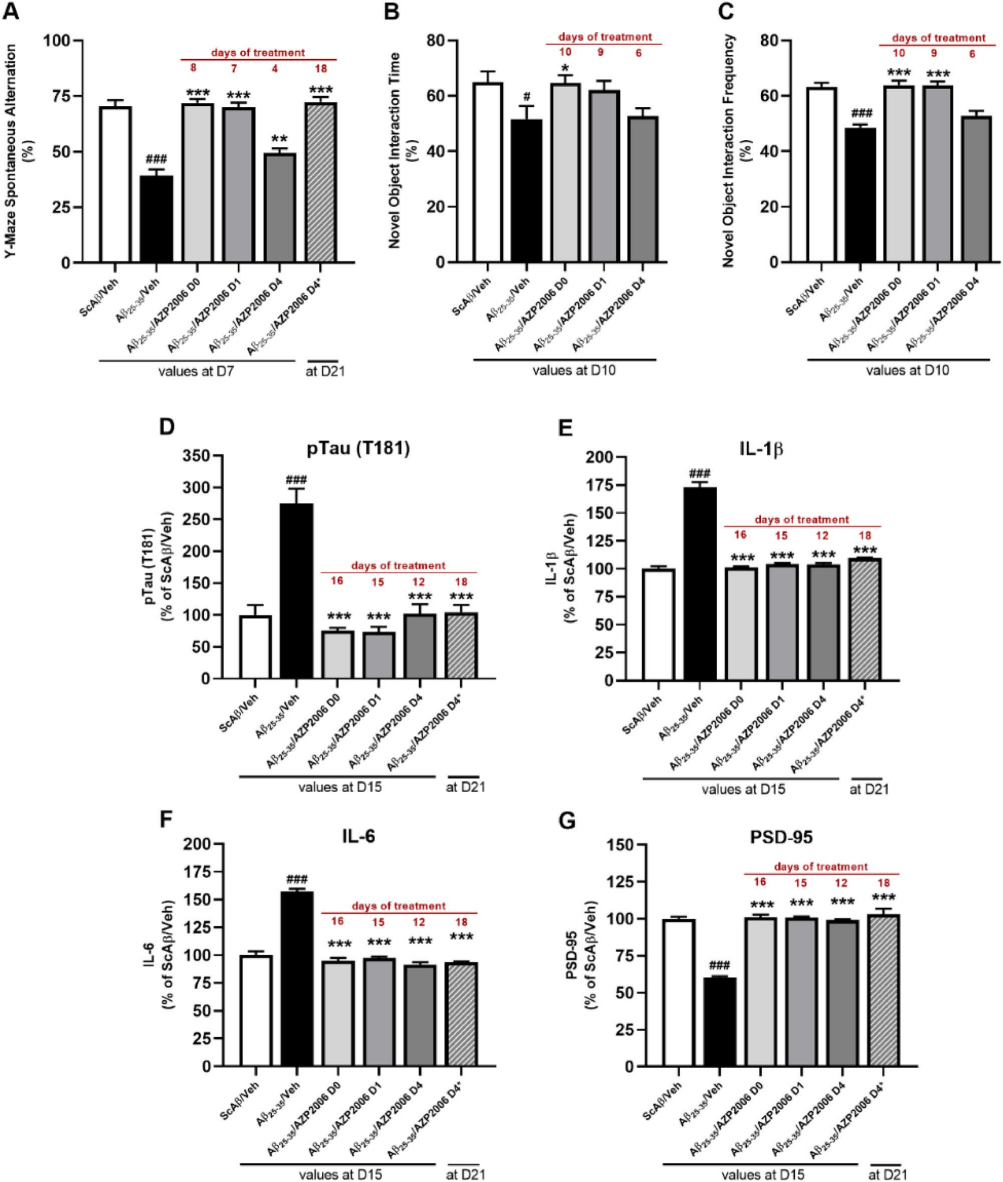
Effect of AZP2006 treatment on Aβ_25–35_ injured mice.** (A**–**C)** Effect of AZP2006 on learning and memory impairments in the C57B/6Rj mice injured by Aβ_25–35_ injection. Mice were daily treated with vehicle (water, D0) or AZP2006 by gavage (2 mg/kg) starting at: day 0 (Aβ_25-35_/AZP2006 D0), day 1 (Aβ_25-35_/AZP2006 D1), day 4 (Aβ_25-35_/AZP2006 D0), and day 4* (Aβ_25-35_/AZP2006 D4*). Treatment duration is shown in red. (**D**) Unilateral *icv* injection of Aβ_25-35_ yielded a significant increase in pTau (T181) level in hippocampus when compared to ScAβ/Veh control mice, which was rescued by AZP2006 treatment. (**E**–**G)** cortex concentrations of IL-1β, IL-6 and PSD-95 proteins measured by ELISA. Results expressed in % of ScAβ/Veh (control group) show the mean ± SEM (standard error of the mean). One-way ANOVA followed by Fisher’s LSD test, n = 10/group. #*p* < 0.01 or ###*p* < 0.001 versus ScAβ/Veh; *p < 0.05, **p < 0.01 or ***p < 0.001 versus Aβ_25-35_/ Veh.

Compared to scramble Aβ (Sc Aβ)-injected mice, Aβ_25–35_-injured mice, showed a significant decrease in percentage of spontaneous alternation (Fig. 4A, black bar) and in the performances at the novel object recognition test (Fig. 4B, C, black bar), confirming the validity of the model. The daily administration of AZP2006 was able to prevent the short-term deficits observed at the Y-maze or NOR tests in a time of exposure-dependent manner (Fig. 4B, C, grey bars). Thus, percentage of alternation, time and number of contacts with objects (frequency) were increased to a level similar to the control one.

At D15 or D21 animals were sacrificed and hippocampus and cortex were used to quantify the levels of phospho Tau (T181), IL-1β, IL-6 and PSD-95. As expected, after Aβ_25–35_ injection, pTau was augmented in the hippocampus (Fig. 4D, black) and levels of IL-1β and IL-6 were significantly increased in the cortex compared with healthy controls (Fig. 4E, F, black). Interestingly, AZP2006 administration was able to decrease all AD markers independently of the treatment duration (Fig. 4A–F, grey), confirming the benefic action previously described in vitro (Fig. 1).

We also evaluated the postsynaptic protein PSD95 level, implicated in synaptic plasticity^27,37^ and found to be reduced in AD patients^38^ and in mice with impaired learning and memory performances^27,39^. Consistently, a 40% reduction of PSD95 expression was measured 16 days after Aβ injury (Fig. 4G, black). Interestingly, AZP2006 treatments were able to rescue PSD95 to normal levels (Fig. 4G, grey). Those levels are well correlated with the spatial memory improvements observed and suggested the existence of a possible mechanism of synaptic induction or synaptic protection where AZP2006 takes a place.

### AZP2006 chronic treatment is able to prevent, to protect and to restore the deficits in SAMP8 mouse model

The SAMP8 (Senescence Accelerated Mouse Prone-8) mouse model is a spontaneous animal model of accelerated aging. Brains of SAMP8 mice have shown age-associated pathologies in the hippocampus including age-related Aβ deposition, impaired Aβ clearance, age-related aberrant hyperphosphorylation of Tau-like neurofibrillary tangles, increased oxidative stress and gliosis as well as deficits in learning and memory making SAMP8 mouse a valuable model to study AD and other cognitive disorders^40,41^. We used this animal model to test the effect of a chronic administration of AZP2006 on AD-like deficits.

**Figure 5:**
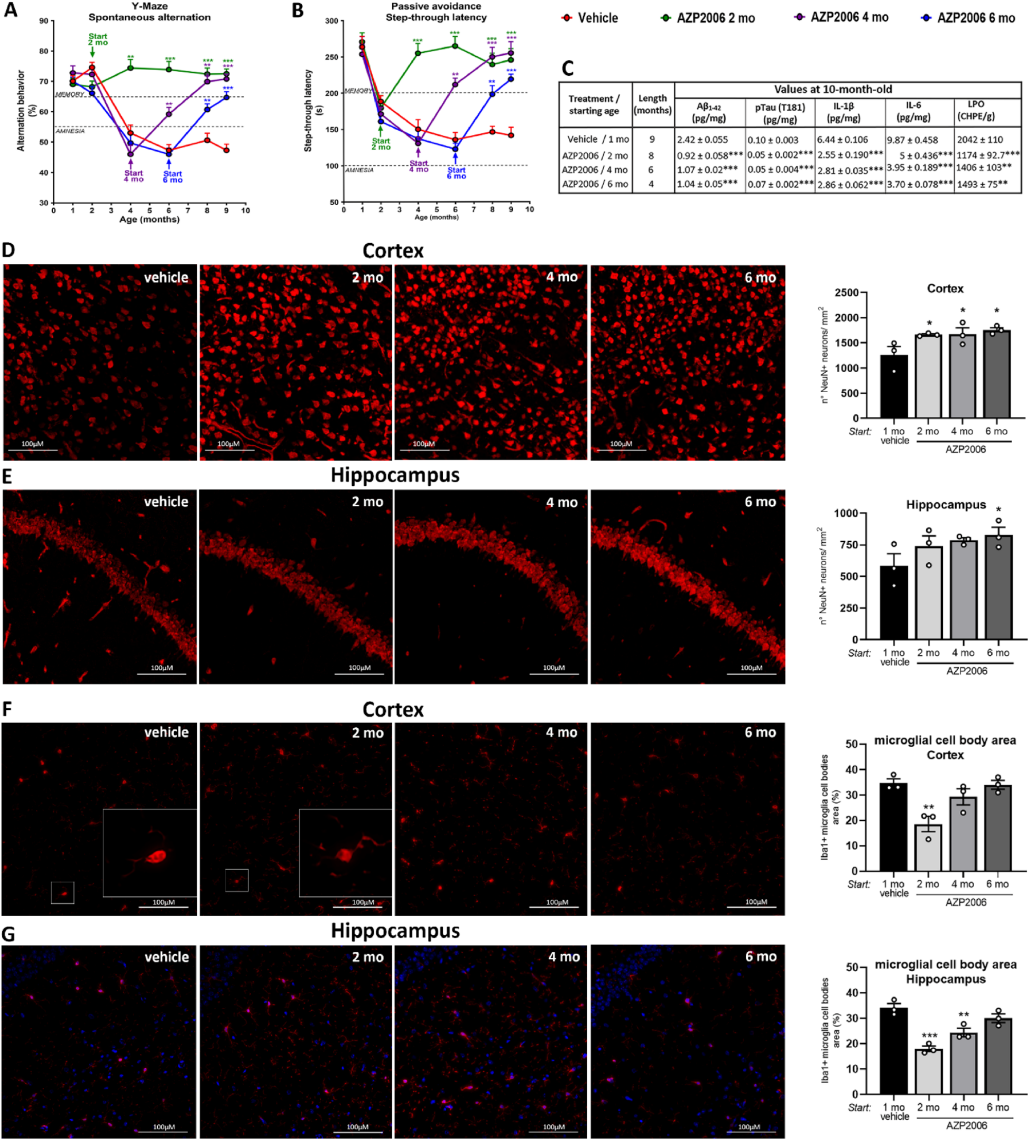
AZP2006 significantly improved senescence behavior and markers in SAMP8 mice.** (A**, **B)** Age-associated behavioral impairments including learning and memory difficulties were measured by Y-maze and Step-Through Passive Avoidance test. Animals received a dose of AZP2006 at 3 mg/kg/day in drinking water for 8, 6 or 4 months or water alone as control (vehicle). The percentage of spontaneous alternation in the Y-Maze was used to measure the exploration of a new environment and Step-Through Passive Avoidance test was used to evaluate recognition memory. (**C**) Aβ_1-42_ and pTau(T181) level (AD markers), cytokines levels (sign of inflammation) and LPO level (oxidative stress) markers normally triggered by aging were reduced upon AZP2006 treatment. The table reports the ELISA quantification of pTau(T181) in hippocampus and Aβ_1-42_, IL-1β and IL-6 in cortex, and the measurement of LPO in hippocampus of 10-month-old SAMP8 mice. All values are expressed as mean ± SEM, n = 10–12/group. **p < 0.01 and ***p < 0.001 versus vehicle. (**D**, **E**) Neuronal cells were identified by immunofluorescence staining with NeuN (red fluorescence) in cortex and hippocampus. *Left*, Representative images of the 4 experimental conditions are shown. *Right,* NeuN quantification was made on 3–9 slides. (**F**, **G)** *Left*, Representative images of Iba1-positive microglia (red fluorescence) in cortex and hippocampus. White squares show a microglia magnification (see also Supplementary Fig. 6). *Right*, Iba1 quantification. One-way ANOVA followed by Fisher’s LSD test. *p < 0.05, **p < 0.01 or ***p < 0.001 versus 1mo vehicle.

As expected, at both behavioral tests, all SAMP8 control animals (vehicle) showed a progressive decline of cognitive abilities (Fig. 5A, B, red line). Thus, SAPM8 rodents rapidly loose the willingness to explore new environments (Y-maze) and the ability to learn and remember the association between the aversive stimulus and the specific environmental context (Passive Avoidance learning).

In this model, AZP2006 treatment induced a significant improvement of all behavioral performances. The chronic administration of AZP2006 (see Supplementary Fig. 4B) significantly increased the percentage of spontaneous alternation at the Y-Maze (Fig. 5A) and the time spent to avoid the electric shock in the Passive Avoidance test (Fig. 5B). When AZP2006 was administered early (at 2 months old, green line), before the appearance of a cognitive decline, it prevented all deficits. At 4-month-old (purple line), AZP2006 administration fully restored cognitive functions. Starting from 6 months of age (4 months after the first cognitive deficits, blue line), AZP2006 partially, but significantly, rescued the cognitive abilities.

AD’s biomarkers analysis was performed at 10 months of age (after 2, 4 or 6 months of treatment). As shown in Fig. 5C, cortical Aβ_1–42_ deposit levels and hippocampal hyperphosphorylated Tau (T181) were decreased after AZP2006 treatment (all periods of treatment). In particular, the effect on Aβ_1–42_ levels was more pronounced in the longer treatment period (0.9 ± 0.058 after 8 months of treatment vs 2.42 ± 0.055 for vehicle group). This improvement was accompanied by an important reduction of cortical proinflammatory cytokines (IL-1β and IL-6), and the Lipid peroxidation marker (LPO) of oxidative stress.

Neu-N positive cells (Neu+) were also immunolabeled in order to quantify neurons number in the cortex (Fig. 5D) and hippocampus (Fig. 5E). By starting AZP2006 treatment at early ages, an increase of ∼40% of neurons were observed in cortex compared with vehicle treated animals. This same pattern was observed across the hippocampal section, even if less evident.

Finally, we used Iba1 immunostaining to visualize morphology of immunoreactive microglia in the cortex (Fig. 5F) and in hippocampus (Fig. 5G). Upon activation, microglia cell body enlarges and branching of processes may either increase (mildly activated) or become swollen or truncated (intermediate activated)^42^. AZP2006 chronic treatment led to an important decrease of microglia activation, reflected in the decrease of size (area) of Iba1 + microglia cell bodies in both cortex and hippocampus (Fig. 5F, G white squares, grey bars in quantification and Supplementary Fig. 6). This effect is consistent with the decreased secretion of proinflammatory cytokines in cortex and the anti-inflammatory role of AZP2006 previously described in vitro.

## Discussion

AZP2006 is a human safe small synthetic polycationic amphiphilic molecule (free base MW = 428.67 g/mol) orally administered and currently under clinical development (Phase 2a clinical trial # NCT04008355). The molecule was proven to have a good absorption, a large exposure and a long half-life (> 15 days in human) with a fast BBB crossing and brain penetration in mice (15 min after an oral administration, AZP2006 was found in different brain areas in particular in the hippocampus).

The present study reports for the first time the neuroprotective effects of AZP2006 against the injuries caused by Aβ oligomers and neurodegeneration linked to aging.

Neuroprotective activity of AZP2006 was firstly investigated in an in vitro coculture of neurons and microglia. In this model, application of Aβ_1–42_ displayed a significant reduction of neuron number, dendritic network and synapses parallel to a severe induction of microglia activity and interleukin release. The addition of AZP2006 together with Aβ_1–42,_ showed neuroprotective effects and microglia deactivation at nM range concentration.

In parallel, the effects of AZP2006 were tested in 2 animal models of AD: an induced mouse model injected with the Aβ_25–35_ fraction of the amyloid peptide and the SAMP8 spontaneous aging model (mutant mouse model of senescence). In both models, the oral treatment with AZP2006 restored the cognition impairments and prevented the central loss of neurons and synapses. We showed that the release of cytokines associated with the massive neuroinflammation were also abolished after the treatment and that the hyperphosphorylation of Tau protein was reduced. These effects were observed for a brain exposure (CSF) of 2 nM concentration of AZP2006 compatible with the active concentrations observed in vitro.

In an attempt to understand the AZP2006 mode of action, we discovered that AZP2006 leads to an augmented level of PGRN and that the artificial PGRN sequestration totally abolishes AZP2006 neuroprotective effects in vitro. In addition, we proved that AZP2006 binds PSAP (the cofactor of PGRN) and inhibits TLR9 receptors normally responsible for proinflammation when activated.

**Figure 6:**
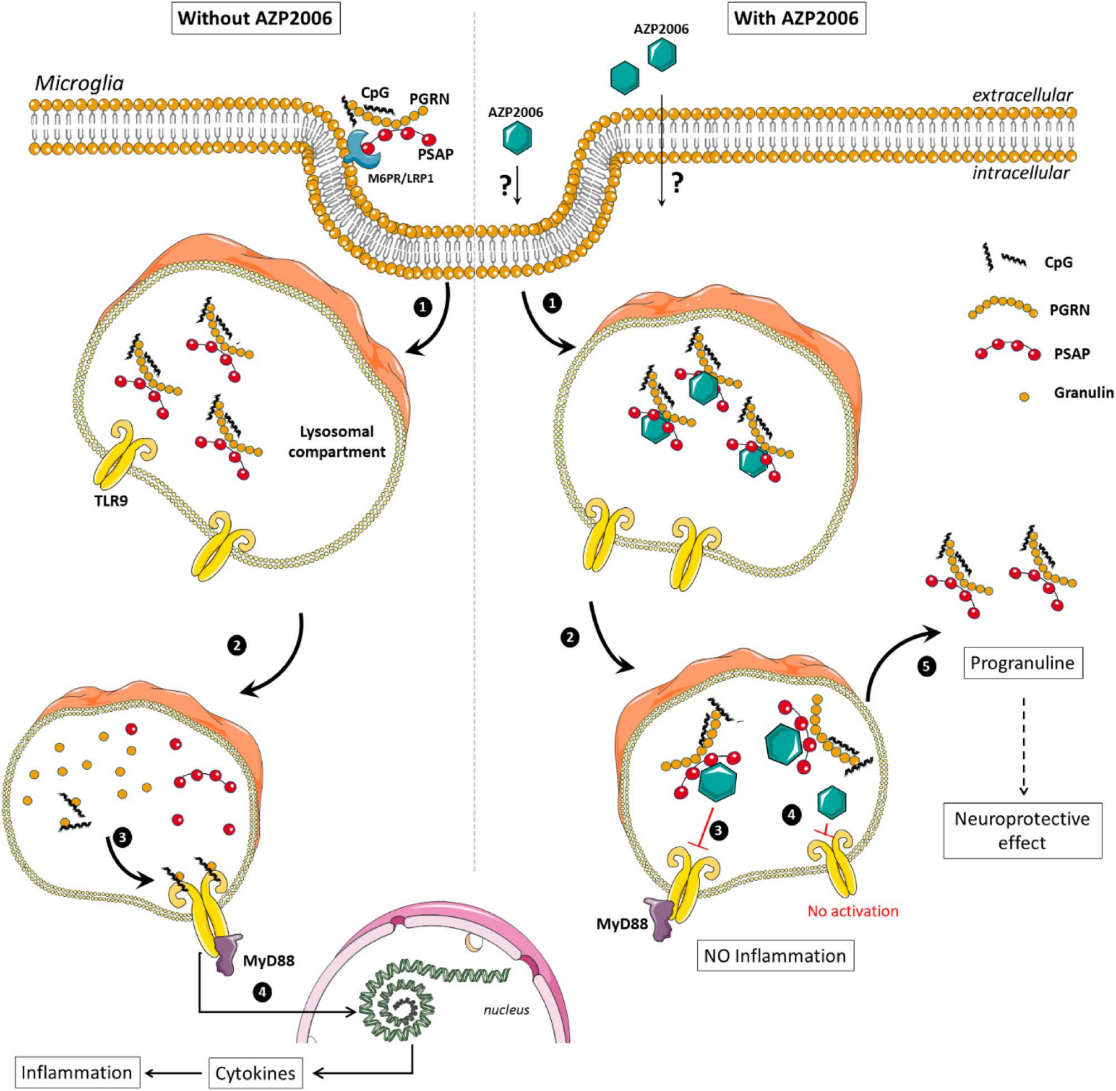
Hypothetical mode of action for AZP2006. Left panel: PGRN targeting is mainly found in microglial cells^44,45^. (**1**) PGRN may be associated with Prosaposin (PSAP) and be carried to endosomes as a passenger via the trafficking receptors M6PR/LRP1^45^. (**2**, **3)** PGRN complexed to PSAP are transferred to lysosomes where PGRN can be subject to protease cleavages that release individual granulin modules in the form of polypeptides of 6 kDa called granulins^78^. Granulin interacts with Toll-like receptor 9 (TLR9) and contribute to its activation^34^. (**4**) Once active, TLR9 triggers MyD88-dependent signaling that induces the production of pro-inflammatory cytokines after activation of NF-kB^79^. Right panel: In presence of AZP2006 (**1**) The mechanism used by AZP2006 to enter the cell is still unknown. However, its localization has been proved to be preferentially within the lysosomes. (**2**) In lysosomes, AZP2006 binds PGRN/PSAP and probably stabilizes the complex impeding PGRN release and cleavage. (**3**) The lack of granulins could be the cause of TLR9 inactivation. (**4**) In parallel, AZP2006 may act as TLR9 receptor direct antagonist, inhibiting its activity and then decreasing the proinflammatory pathway activation. (**5**) AZP2006, by preventing the processing of PGRN, can increase its release, which in turn could mediate the neuroprotective effects.

PRGN is a highly conserved secreted protein that is expressed in multiple cell types, both in the central nervous system (CNS) and in peripheral tissues^12,43^. It regulates cell growth, survival, repair and play a major role in regulation of lysosomal function and microglial responses in the CNS. Although its function in lysosome remain to be fully elucidated, PGRN delivery into lysosomes can be driven by the sortilin receptor^44^ or the PSAP-M6PR/LRP1 receptor complex^32,45^. Once in the lysosome, PGRN can be cleaved into individual granulin (GRN) peptides^12^and PSAP into individual saposins (A, B, C, and D) which in turn, serve as activators of lysosomal sphingolipid metabolizing enzymes^46^. Once released, GRN are cofactors that potentiate TLR9-driven signaling and pro-inflammatory responses^34^ (Fig. 6).

Due to its polycationic amphiphilic features, AZP2006 has a high affinity for the lysosome vesicles (LAMP1 positive) and was proven to accumulate into these organelles.

Here, we showed that AZP2006 is able to strongly bind the complex PGRN/PSAP which suggests a mechanism where the accumulation of AZP2006 in lysosome and its interaction with PGRN/PSAP physically blocks the PGRN processing and degradation into GRN with the final reduction of GRN-driven inflammation and cytokines release (Fig. 6).

It is accepted with the AD Literature that neuroinflammation contributes to the exacerbation of AD pathology. This involves an initial inflammatory stimulus (cellular debris, amyloid deposits…) triggering activation of microglia, the resident immune cells of the CNS^47–49^. In this cell type, all TLRs are expressed^50^ and their expression is depending on various stimuli, including Aβ^51^. Several of these receptors reside intracellularly, including TLR9, which is primarily located at the endoplasmic reticulum and translocated to the lysosomal compartment in presence of pathogenic molecules and stress^52^. Once activated, TLR9 triggers an early proinflammatory cytokine response, oxidative stress and inflammation^50^. In our study, AZP2006 antagonizes the TLR9 activation. This result nicely correlates with the reduction of the pathological release of IL-1β and IL-6.

It is worth noting that AZP2006 treatment leads to an augmented level of PGRN. Given the role of PGRN as a protective neurotrophic factor, we cannot exclude that the majority of the beneficial effects of AZP2006 may be mediated through this activity.

Indeed, PGRN can stimulate cell division, invasion, survival and vessel growth in vitro^53^, in vivo^54^ and plays a critical role as wound-related growth factor^55^. Mechanistically, PGRN stimulate MAP kinase (ERK1/2 ) activity^56,57^, which is a vital mediator of a number of cellular fates including growth, proliferation, and survival^58^ while it activates (PI3K)/Akt cell survival pathways, which are critical in rescuing neurons from cell death induced by glutamate or oxidative stress^59^. Finally, PGRN enhances GSK-3β phosphorylation ((PI3K) activity-dependent) causing neural progenitor cell proliferation^60^. GSK-3β phosphorylation, synonymous of GSK-3β inactivation, may explain the large reduction of hyperphosphorylated Tau protein observed after AZP2006 treatment both in vitro and in vivo. Nevertheless, the effect of AZP2006 on tau hyperphosphorylation persists even in partial loss of PGRN, suggesting the existence of additional pathways (PGRN-independent) involved in AZP2006 mechanism of action.

The relationship between PGRN and neurodegenerative diseases has been widely described. Thus, GRN gene haploinsuffiency was found to cause frontotemporal lobar degeneration (FTLD)^16,61,62^. An homozygous-null GRN mutation carrier has been reported presenting a clinical phenotype similarity to the neuronal ceroid lipofuscinosis (NCL), a lysosomal storage disease^63^. Also, mutations in GRN gene have been identified in cohorts of clinically diagnosed AD^64^, PD^65^ and PSP patients^66^. Therefore, strategies to increase PGRN levels in patients could provide an effective treatment in neurodegenerative diseases. AZP2006 is a highly innovative and promising molecule totally inscribed in this context.

In parallel, we saw a progressive decrease of TLR9 activity by increasing AZP2006 concentration. Interestingly, such inhibition was not rescued by the presence of ODN, CpG oligonucleotides that strongly activate TLR9 receptor by direct binding^67^. Therefore, a possible interaction between AZP2006 and TLR9 should not be excluded.

Given the multiple actions of AZP2006, more experiments are needed to clearly understand the mechanism underpinning PGRN release and TLR9 inhibition. In particular, it would be interesting to better identify the mechanism used by AZP2006 to enter the cell and to target the lysosomes.

In summary, we proved here that AZP2006 protects neurons in vitro and improves behavior in vivo models similar to AD pathology. In addition, these effects seem involving, at least partially, PGRN factor.

In light with these results, AZP2006 could be considered as a potential candidate for the treatment of AD and related disorders. Nevertheless, some additional pre-clinical experiments on transgenic AD models (Tg2576 or 3xTg-AD) are needed.

Today, AZP2006 is in clinical trial in PSP patients in a phase 2a study in order to better investigate the pharmacokinetics over a 3 month-period of treatment. In those patients, PGRN levels in CSF will be evaluated together with biomarkers for disease progression (NfL, Aβ, pTau and cytokines).

## Materials and methods

### Culture of cortical neurons

Rat or mice cortical neurons were cultured as previously described^24,68^ and adapted to include microglial cells. Briefly pregnant female rats or mice (Wistar and Swiss, Janvier labs, Le Genest-Saint-Isle, France) were killed using a deep anesthesia with CO2 chamber and a cervical dislocation at 15 days and 14 days of gestation respectively. Then, fetuses removed from the uterus and immediately placed in ice-cold L15 Leibovitz medium with 2% penicillin (10,000 U/ml) and streptomycin (10 mg/ml) solution (PS) and 1% bovine serum albumin (BSA).

Cortices were treated for 20 min at 37 °C with a trypsin-EDTA solution at a final concentration of 0.05% trypsin and 0.02% EDTA. The dissociation was stopped by addition of Dulbecco’s modified Eagle’s medium (DMEM) with 4.5 g/liter of glucose, containing DNAse I grade II (final concentration 0.5 mg/ml) and 10% fetal calf serum (FCS).

Cells were mechanically dissociated by three forced passages through the tip of a 10-ml pipette. Cells were then centrifuged at 515×*g* for 10 min at 4 °C. The supernatant was discarded, and the pellet was resuspended in a defined culture medium consisting of Neurobasal medium with a 2% solution of B27 supplement, 2 mmol/liter of L-glutamine, 2% of PS solution, 10 ng/ml of brain-derived neurotrophic factor (BDNF), 2% of heat-inactivated horse serum, 2% of heat-inactivated FCS, 1 g/L of glucose, 1 mM of sodium pyruvate, and 100 μM of non-essential amino acids. Viable cells were counted in a Neubauer cytometer, using the trypan blue exclusion test. Cells were seeded at a density of 45,000 per well in 96-well plates precoated with poly-L-lysine for immunostaining and 25,000 per well in 96-well plates for siRNA transfection. Cells were cultured at 37 °C in an air (95%)-CO_2_ (5%) incubator and culture medium was changed every day.

### Human Aβ_1–42_ and compounds exposure

Cortical neurons and microglia were incubated with Aβ solutions after 11 days of culture. The Aβ_1–42_ preparation was done following the procedure described by^24^. Briefly, Aβ_1–42_ peptide (Bachem, Weil-am-Rhein, Germany) was dissolved in the defined culture medium mentioned above, at an initial concentration of 40 μmol/L. This solution was gently agitated for 3 days at 37 °C in the dark and immediately used after being properly diluted in control medium to the concentrations used (5 μM of Aβ_1–42_ preparation containing 0.5 μM of Aβ oligomers (AβO) measured by WB). The control medium consisted in the defined culture medium (as described above).

AZP2006 and anti-PGRN (5 μg/mL, MAB2557, R&D Systems, Noyal Châtillon sur Seiche, France) were dissolved in culture medium and incubated with Aβ_1–42_ preparation on primary cortical neurons for 72 h.

### siRNA transfection and Glutamate injury

On day 12, the cultures were transfected with the siRNA CT (control mismatch, SIC001-10NMOL, Sigma-Aldrich Chemie, Saint Quentin-Fallavier France) or with the siRNA target (PGRN, PSAP or PGRN + PSAP) at 20 nM (L-090442-02-0010, L-080037-02-0010, Dharmacon, Horizon Discovery Group plc, Cambridge Research Park, United Kingdom). The transfection was performed with the kit INTERFERin® (POL409-10, Polyplus transfection, Ozyme, Saint-Cyr-l'École, France) (see also Supplementary Fig. 3B).

On day 13 of culture, the cortical neurons were exposed to glutamate at final concentration of 20 µM (diluted in control medium) in presence of AZP2006 for 20 min. After 20 min, glutamate was removed and fresh culture medium with AZP2006 was added for additional 48 h.

### Immunostaining

After Aβ_1–42_ intoxication, cortical neurons were fixed by a cold solution of ethanol (95%) and acetic acid (5%) for 5 min at − 20 °C. Cells were then permeabilized and non-specific sites blocked with a solution of phosphate buffered saline (PBS) containing 0.1% of saponin and 1% FCS for 15 min at room temperature.

Then, cells were incubated for 2 h with the chicken polyclonal antibody anti microtubule-associated-protein 2 (MAP-2, Abcam, Cambridge, USA); the mouse monoclonal antibody anti-SIRP alpha/CD172a (OX-41, Novus, Lille, France); the mouse monoclonal anti-tau phosphor (Thr212, Ser214) AT-100 (Thermo Fisher Scientific, Illkirch-Graffenstaden, France); the mouse PSD95 monoclonal antibody (Abcam, Cambridge, USA); the polyclonal antibody anti-synaptophysin (SYN, Abcam, Cambridge, USA). These antibodies were labeled with Alexa Fluor secondary antibodies (Thermo Fisher Scientific) at the dilution of 1/400 in PBS containing 1% FCS, 0.1% saponin, for 1 h at room temperature. For each condition, between 30 and 40 pictures per well were taken using ImageXpress (Molecular Devices, San Jose, USA) with 20× magnification. All images were generated using the same acquisition parameters and analyses were directly and automatically performed by using Custom Module Editor (Molecular Devices) (Supplementary Fig. 7).

### BDNF quantification

The quantification of extracellular BDNF was performed with a BDNF ELISA kit (ab213899, Abcam, Cambridge, USA) 72 h after intoxication.

### PGRN quantification

PGRN quantification was performed on the cell supernatant (extracellular levels) with a PGRN ELISA kit (AG-45A-0043YTP-KI01, COGER SAS, Paris, France), according to manufacturer’s recommendations 72 h after intoxication.

### IL-1 β and IL-6 evaluation

The amounts of IL-1 β and IL-6 released by the cells were measured by using the following kits: IL-6 rat ELISA Kit (BMS625), IL1B rat (BMS630), IL-6 mouse (KMC0061) and IL1B mouse (BMS6002) (all purchased by Thermo Fisher Scientific, Illkirch-Graffenstaden, France).

### Sample preparation/protein total evaluation

Cells were lysed with CelLyticMT reagent (Sigma-Aldrich Chemie, Saint Quentin-Fallavier France) according to manufacturer’s recommendations. For each condition, the quantity of protein was determined by Pierce kit BCA (Thermo Fisher Scientific, Illkirch-Graffenstaden, France).

### MicroScale thermophoresis (MST)

Both PGRN Protein (SInoBio cat # 10826-H08H) and PSAP (SinoBio cat # 16224-H08H) at a concentration of 3.96 μM were labeled using Monolith™ Protein Labeling Kit RED-NHS (NanoTemper Technologies, Munich, Germany) according to the manufacturer’s instructions in its storage buffer (1 × PBS pH 7.4, 5% Trehalose, 5% Mannitol, 0.01% Tween-80). After labeling, the protein was eluted into binding buffer (1 × PBS pH 7.4, 0.05% Pluronic F-127), which was also used as assay buffer for MST experiments. The concentration of protein after labelling was 444 nM. MicroScale Thermophoresis (MST) binding experiments were carried out with 3 nM NT647-labeled PGRN in binding buffer with a range of concentrations of AZP2006 (0.9155–30,000 nM) at 40% MST power, 15% LED power in premium capillaries on a Monolith NT.115 pico device at 25 °C (NanoTemper Technologies, Munich, Germany). Data was analyzed using NT. Analysis Analysis software (version 1.5.41, NanoTemper Technologies) at the standard MST-on time of 10 s (respectively Thermophoresis + TJump). No aggregation effects or unspecific adhesion of the protein to the glass surfaces of the capillaries were detected. Two technical runs were performed. Data did not possess amplitudes > 5 units combined with Signal to Noise levels > 5 units and was hence defined as non-binder. PSAP protein binding experiments were carried out with 3 nM NT647-labeled PSAP in binding buffer with a range of concentrations of AZP2006 (0.9155–30,000 nM) or PGRN (0.0604–1978 nM).

### Effect on hTLR-9 receptor

Samples and controls were tested in triplicate on recombinant HEK-293 cell line (InvivoGene; HEK-Blue™ hTLR9 cells). This cell line functionally over express hTLR9 protein as well as the alkaline phosphatase gene driven by a NFkB inducible promoter. A recombinant HEK-293 cell line expressing only the reporter gene (alkaline phosphatase) was used as negative control.

HEK 293-hTLR9 cell line was incubated with AZP2006 (3, 10, 30, 100, 300 and 1000 nM) for 60 min prior to a stimulation with the specific agonist ODN 2006 (300 ng/ml) in a 200 μl final reaction volume for 18 h. The synthetic oligonucleotide ODN TTAGGG (A151) (at 3 μg/ml) was used as antagonist positive control. hTLR9 activation results are shown as optical density values (OD).

### Aβ_25–35_ mouse model

Females C57B/6Rj mice (n = 10/group) of 5 weeks old (Charles River, L'Arbresle, France) were kept for housing and experiments at Amylgen's animal facility (Montferrier-sur-Lez, France). Animals were housed in groups with access to food (#A04C, Safe Diet, Augy, France) and water ad libitum, except during behavioral experiments. They were kept in a temperature and humidity-controlled animal facility on a 12 h/12 h light/dark cycle (lights off at 07:00 pm). All animal procedures were conducted in strict adherence to the European Union Directive of September 22, 2010 (2010/63/UE). AZP2006 was dissolved in sterile bidistilled water before each administration. AZP2006 was administered at a dose of 2 mg/kg (free base equivalent/Active Pharmaceutical Ingredient).

Females were daily treated with vehicle (water) by gavage, from D0 (day of Aβ_25–35_ injection) to D15. AZP2006 was administrated from D0, D1 or D4 to D15 or D21 (AZP2006/ Aβ_25–35_ D4*).

On D0, a homogeneous oligomeric preparation of the Aβ_25–35_ peptide (9 nmol/mouse, Genepep, Saint-Jean-de-Védas, France) or Sc. Aβ peptide (9 nmol/mouse, Genepep) was administered to mice in a final volume of 3 µl/mouse, according to the previously described method^35,69–71^.

Mice were anesthetized 5 min with 2.5% isoflurane, restrained and the head immobilized, then were injected into right lateral ventricle of the brain through a 28-gauge stainless-steel needle, 4 mm long. An injection volume of 3 μl was delivered gradually within 30 s and the needle left in place for an additional 30 s before being removed^72^. The treatment efficacy was evaluated on the improvement of the Aβ_25–35_-induced learning and memory deficits in spatial working memory (spontaneous alternation in the Y-maze) at D7 or D21, and in spatial learning and memory (NOR test) with 3 sessions from D08 to D10.

### Novel object recognition (NOR) test

From D08 to D010, all mice performed novel object recognition test to measure recognition memory. In this test, the activity was analyzed using the nose tracking protocol, in terms of number of contacts with objects (frequency) and duration of contacts (time). Novel object recognition (NOR) procedure was recorded by Videotrack software (Viewpoint) and filmed. The task procedure consists of three sessions.

Session 1: mice were placed individually in a squared open-field (50 cm × 50 cm × 50 cm high) made of white plexiglass and a floor equipped with infrared light emitting diodes. Mice were habituated to the open-field during a 10-min duration session and their locomotor activity captured through an IR-sensitive camera and analyzed using the Ethovision® software (Noldus). The activity was analyzed in terms of locomotor speed (cm/s), percentage of presence in the central area (25 × 25 cm) defined by the software and stereotypes (rearing and grooming).

Session 2: 24 h after session 1, two identical objects (50 ml plastic vials with caps) were placed at defined positions (at two opposite edges of the central area). Each mouse was placed in the open-field and the exploratory activity was recorded for 10-min. The activity was analyzed using the nose tracking protocol, in terms of number of contacts with objects (frequency) and duration of contacts.

Session 3: 24 h after session 2, the object in position #2 was replaced by a novel one (a soft plastic chair feet protection) differing in color shape and texture from the familiar object. Each mouse was placed again in the open-field and the exploratory activity was recorded during a 10-min session. The activity was analyzed like in session 2. The preferential exploration index was calculated as the ratio of the number (or duration) of contacts with the object in position #2 over the total number (or duration) of contacts with the two objects. Animals showing less than 10 contacts with objects during the session 2 or session 3 were discarded from the study. Usually, it represents 10–15% of the animals (high attrition test). The videotrack system can follow 4 animals simultaneously. Animals were tested according to their numeration. Between two sessions the open-field and objects were cleaned between two sessions using water followed by 50% ethanol solution. On D15 or D21 all mice were anesthetized by 200 µL intraperitoneal injection of a premix of ketamine (80 mg/kg) and xylazine (10 mg/kg). All mice were sacrificed by decapitation and hippocampus and cortex were used for biomarkers quantification.

### Senescence-accelerated mouse-prone 8 (SAMP8) mouse model

SAMP8 female mice (n = 10–12/group), 4–5 weeks old (Envigo, Gannat, France), were kept for housing and experiments at Amylgen's animal facility (Montferrier-sur-Lez, France). Animals were housed in groups with access to food (#A04C, Safe Diet, Augy, France) and water ad libitum, except during behavioral experiments. They were kept in a temperature-controlled animal facility on a 12 h/12 h light/dark cycle (lights off at 07:00 pm). Mice were identified by a chip implanted subcutaneously. All animal procedures were conducted in strict adherence to the European Union Directive of September 22, 2010 (2010/63/UE). Animals were placed into Innocage® IVC Rat Caging System at n = 4–6 animals per cage. Mice were daily treated p.o. (drinking water) with vehicle or 3 mg/kg/day AZP2006 from the age of 1 (vehicle), 2, 4 or 6 months (AZP2006) to 10 months. Treatment solutions were freshly prepared once a week before administration and were kept protected from light. The treatment efficacy was evaluated on the improvement of the spontaneous SAMP8 learning and memory deficits in spatial working memory (spontaneous alternation in the Y-maze) and in the contextual long-term memory (step-through passive avoidance task) at 1, 2, 4, 6, 8 and 9 months of age.

### Y-maze test

The Y-Maze apparatus was designed according to^73,74^. Each arm is 40 cm long, 13 cm high, 3 cm wide at the bottom, 10 cm wide at the top, and converging at an equal angle. Each mouse was placed at the end of one arm and allowed to freely move through the maze during an 8 min session. The series of arm entries, including possible returns into the same arm, were checked visually by an experimenter blind to treatment. An alternation was defined as entries into all three arms on consecutive occasions. The number of maximum alternations is therefore the total number of arm entries minus two and the percentage of alternation was calculated as (actual alternations/maximum alternations) × 100. Parameters included the percentage of alternation (memory index) and total number of arm entries (exploration index)^35,69,70^. No animals showing an extreme behavior (alternation percentage < 20% or > 90% or number of arm entries < 8) were discarded from the calculation.

### Step-through passive avoidance test

The apparatus was a two-compartment (one white and one black) polyvinylchloride walls box (15 × 20 × 15 cm high), with a grid floor and a guillotine door that separates each compartment. A 60 W lamp positioned 40 cm above the apparatus lights up the white compartment during the experiment. Whereas one is illuminated (white) the other is darkened (black). Scrambled footshocks (0.3 mA for 3 s) can be delivered to the grid floor using a shock generator scrambler (Med Associates, Fairfax, USA). The guillotine door was initially closed during the training session. Each mouse was placed into the white compartment. After 5 s, the door was raised. When the mouse entered the darkened compartment and placed all its paws onto the grid floor, the door was closed and the footshocks delivered for 3 s. The step-through latency, that is, the latency spent to enter the darkened compartment, and the subjective frequency and intensity of vocalizations were recorded manually by an experimenter blind to treatment. The retention test was carried out 24 h after training. Each mouse was placed again into the white compartment. After 5 s, the door was raised. The step-through latency was recorded up to a cut-off time of 300 s^35,69,70^.

### Immunohistochemistry

Before tissues dissection, mice were anesthetized by 200 µL intraperitoneal (IP) injection of a premix of ketamine (80 mg/kg) and xylazine (10 mg/kg).

The brains of 10-month-old SAMP8 mice were transcardially perfused with saline solution. Half-brains (right hemisphere) were post-fixed in with 4% paraformaldehyde in 0.1 M PBS, pH 7.4 for 48 h and cryoprotected PBS 15% sucrose for 48 h at 4 °C and then in PBS 30% sucrose at 4 °C until embedded in Tissue-Tek® O.C.T.™ (Sakura, Villeneuve d’Ascq, France).

Cryostat Sects. (7 µm) were collected in PBS at 4 °C, mounted on superfrost slides (VWR Superfrost +), air dried, and subjected to fluorescent staining. For each brain, sections were collected at the level of the anterior, medial and posterior hippocampus (Bregma − 1.46 mm, − 1.94 mm or − 2.46 mm respectively, Paxinos and Franklin’s mouse brain atlas-replace by reference number). One of each section by level (3 sections/brain) were placed onto the same slide.

Fluorescence immunostaining was performed with an automate Roche® (Discovery XT or Ultra-Ventana, Rotkreuz Switzerland). Slides were incubated or not with mouse anti-NeuN antibody (1/4000; #MAB377, Millipore, Burlington, USA) or goat anti-Iba1 antibody (1/1000; #NB100-1028, Biotechne, Minneapolis, USA) for 60 min at 37 °C. The reactions were developed by incubation at 37 °C with a secondary antibody anti-mouse IgG (ready to use; Discovery OmniMap #760-4310, Ventana Medical Systems, Tucson, Arizona, USA) for 16 min or with rabbit anti-Goat IgG (H + L) (1/500; #6160-01, Southern Biotech, Birmingham, AL, USA) for 32 min followed by an incubation with the Discovery Rhodamine Kit (RUO) (ready to use; #760-233 Ventana Medical Systems, Tucson, Arizona, USA) for 20 min at 37 °C. After washing, the slides were incubated with 500 µl of 4′, 6‐diamidino‐2‐phenylindole (DAPI) (1/500; #6244, Eurobio, Les Ulis, France) for 5 min at RT in the dark to stain nuclei and mounted with Fluoromount-G® (Clinisciences, Nanterre, France).

The immunofluorescent slides were digitized at 20 × magnification (resolution of 0.6484 µm/pxl) using a PathScan® scanner (Excilone, Élancourt, France) in immunofluorescence. The 3 sections of each slide were entirely scanned into 3 separated regions of interest (ROI). The imaging and quantification were performed with the Halo® Imaging Analysis software (IndicaLabs; Corrales, NM, USA). Halo® measures and reports individual cell data maintaining an interactive link between cell metrics and cell imagery. The number of NeuN positive neurons, the Iba1 positive microglia and its body-cell area in a section of the cortex and in the whole hippocampus were automatically counted with the HALO software (Supplementary Fig. 6A). Halo® platform is designed to be used through modules which are pre-built algorithms designed for a particular image analysis. The Cytonuclear FL module was used to measure the immunofluorescent positivity of NeuN and Iba1 fluorescent marker (TRITC channel) in a radius around the nuclei of 0.5 µm for NeuN (entire cell, meaning nucleus and cytoplasmic area) and 1.5 µm for Iba1 (from the nuclei borders into the TRITC channel, this radius allowed to consider the Iba1 positivity of the arborization of activated microglia and so to detect and count the activated microglia). The DAPI channel was used to detect and segment the nuclei. A mean intensity value was determined as positivity threshold. The Object Colocalization FL v1.0 module was used to determine the intensity threshold values between Iba1 positive microglia cell bodies and its arborization (Supplementary Fig. 6B–D).

### Biomarkers quantification by ELISA

After thawing, the cortex and hippocampus were homogenized in 50 mM Tris-150 mM NaCl buffer, pH 7.5, and sonicated for 20 s. After centrifugation (16,100 g for 15 min, 4 °C), supernatants were used for ELISAs assays according to instructions of their respective manufacturer. Phospho Tau (T181, #KOH0613, Novex, Thermo Fisher Scientific, Illkirch-Graffenstaden, France), content measurement was performed in hippocampus. Amyloid beta_1–42_ (Aβ_1–42_, #CEA946Mu, Cloud-Clone Corp., Park Row, USA), Interleukin 1β (IL-1β, # SEA563Mu, Cloud-Clone Corp., Park Row, USA), Interleukin 6 (IL-6, #1857233, Thermo Fisher Scientific, Illkirch-Graffenstaden, France) and postsynaptic density (PSD95, #SEG168Mu, Cloud-Clone Corp., Houston, USA) content measurements were performed in cortex.

For all assays, absorbances were read at 450 nm and each samples concentration was calculated using the standard curve. All results were expressed in % of scrambled (ScAβ/Vehicle). All samples were assayed in duplicate and the average of these duplicates were used for calculi.

### Lipid peroxidation measurement (modified FOX assay)

For LPO measurement hippocampi from each group were used as described by^75^. After thawing, tissues were homogenized in cold methanol (1/10 w/v), centrifuged at 1,000 g during 5 min and the supernatant placed in Eppendorf tube. The reaction volume of each homogenate was added to FeSO_4_ 1 mM, H_2_SO_4_ 0.25 M, xylenol orange 1 mM and incubated for 30 min at room temperature. After reading the absorbance at 580 nm (A5801), 10 µl of cumene hydroperoxyde (CHP) 1 mM were added to the sample and incubated for 30 min at room temperature to determine the maximal oxidation level. The absorbance was measured at 580 nm (A5802). The level of lipid peroxidation was determined as CHP equivalents according to: CHPE = A5801/A5802 x [CHP (nmol)] and expressed as CHP equivalents per wet weight of tissue and as percentage of control group data (Vehicle-treated mice).

### Data analysis and statistics

Results are expressed as mean ± SEM (standard error of the mean). In vitro studies statistical analyses on the different conditions were performed using ANOVA followed by Dunnett’s or Fisher’s t test. In vivo statistical analyses were performed on the different conditions using two-way ANOVA (F value), followed by the Dunnett’s post-hoc multiple comparison test. Passive avoidance latencies do not follow a Gaussian distribution, since upper cut-off times are set. They were therefore analyzed using a non-parametric method the Kruskal–Wallis H test (two-way ANOVA on ranks), followed by a Dunn's multiple comparison test. *p* < 0.05 was considered as statistically significant. All test were performed by using GraphPad Prism software version 9.0.2.

### Ethics statement

Experiments were performed by authorized laboratory (agreement #A 34-169-002 from May 02, 2014) after approval by the ethic committee of Direction Régionale de l'Alimentation, de l'Agriculture et de la Forêt du Languedoc-Roussillon and by the Ministry of Higher Education and Research (#02441). They followed current European Union regulations (Directive 2010/63/EU). The results were reported according to The Animals in Research: Reporting in vivo Experiments Guidelines—ARRIVE^76^.

## Supplementary Information

Supplementary Information.
