# Supplementary material for: AZP2006, a new promising treatment for Alzheimer’s and related diseases
Source: Sci Rep. 2021 Aug 19;11:16806. doi: 10.1038/s41598-021-94708-1 (PMC8376949; doi:10.1038/s41598-021-94708-1)
Supplement: Supplementary file 1 — Supplementary Information. [file 41598_2021_94708_MOESM1_ESM.docx]

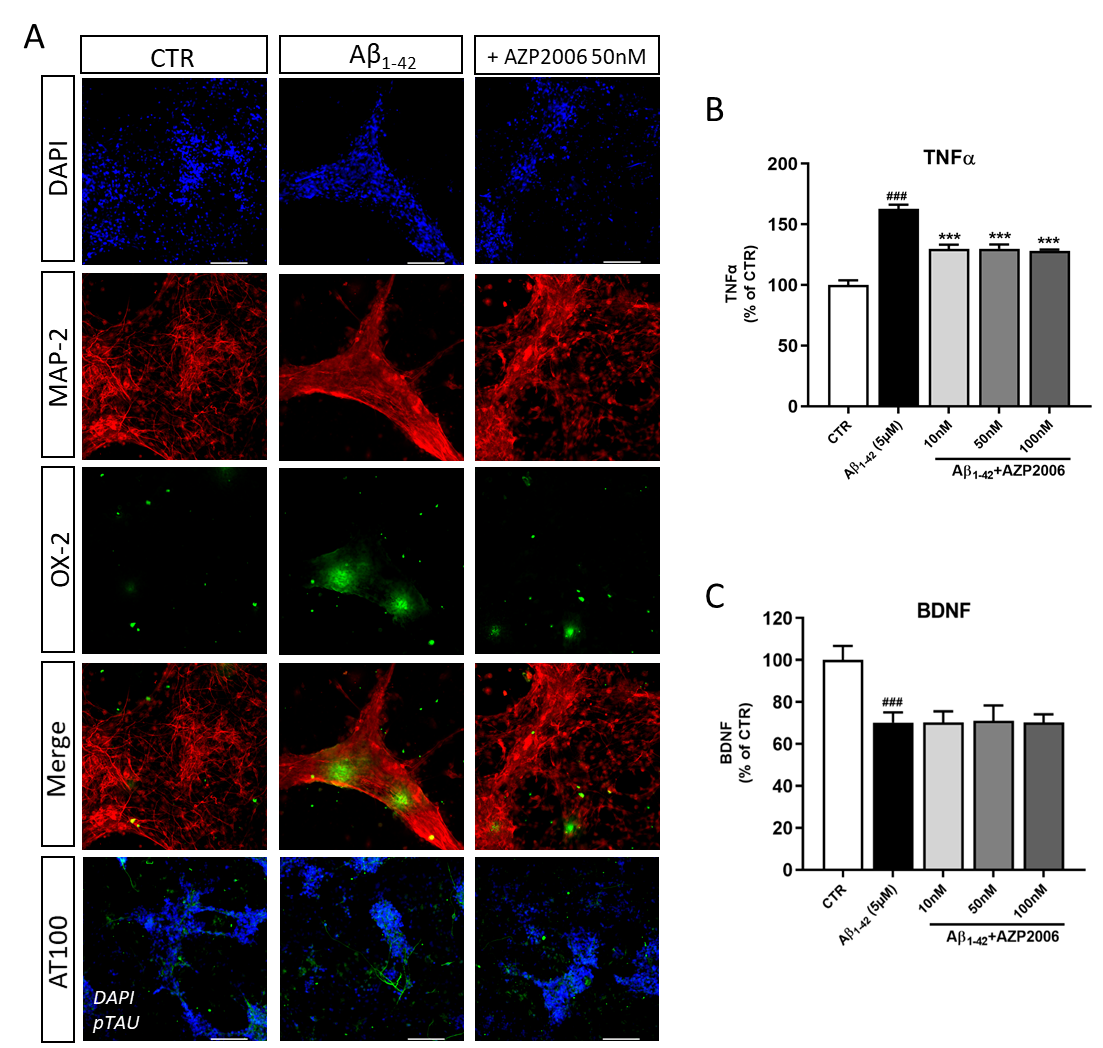


**Supplementary Figure 1:** **A**, representative pictures associated to the quantification in Figure 1 of the main text. **B**, effect of AZP2006 on TNFα release in primary rat culture of cortical neurons cultured with microglia and injured with Aβ peptides for 72h (5 µM corresponding to 0.5 µM of AβO). Control mean is 0,08 pg/ml/cell, +/- 0,003, n = 5 (CTR = no β amyloid, no compound). One-way ANOVA followed by PLSD Fisher’s test, n=5-6. *p<0.05 vs CTR. #p<0.05 vs Aβ1-42 condition. **C**, Measurement of extracellular BDNF level in primary cortical neurons and microglia co-cultures intoxicated with Aβ solutions (72 hours). Results are expressed in mg/mL after normalization by the total protein level of the cell culture. Control mean is 24.72 ng/ml, +/- 1.66, n = 6. Data are presented as means ± SEM of 6 determinations. One-way ANOVA followed by PLSD Fisher’s test. # p< 0.05 vs. control

**
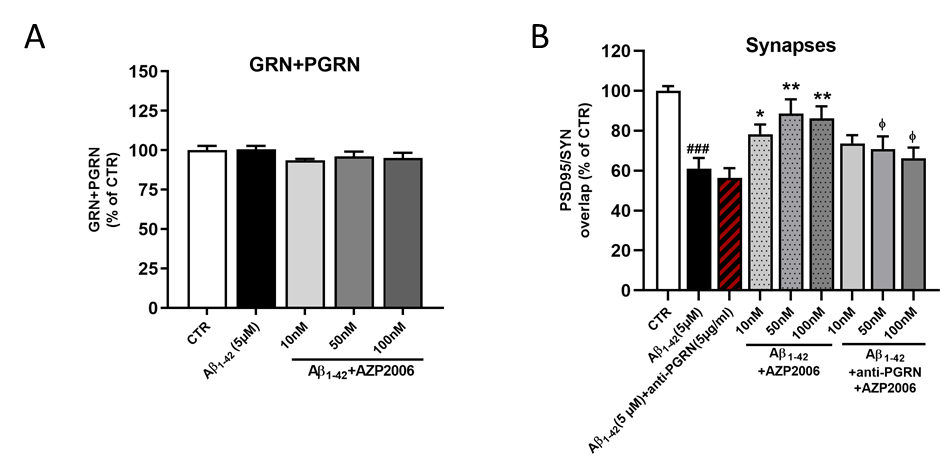
**

**Supplementary Figure 2: A,** effect of AZP2006 on progranulin and granulin cytosolic levels in primary cortical neurons cultured with microglia injured by Aβ (5µM, 72h). Control mean is 5.50 ng/ml, +/- 0.06, n = 6. One-way ANOVA followed by PLSD Fisher’s test**.** AZP2006 doesn’t modify the intracellular PGRN and GRN concentrations. **B,** Synapses number automatically counted by the Custom Module Editor (from Molecular Devices). The PSD95/SYN overlapping (µm²) was considered as indicator for an active synapse. CTR = 526.7 µm^2^/well, +/- 12.3, n = 4. All values are expressed as mean +/- SEM (standard error of the mean). One-way ANOVA followed by PLSD Fisher’s test, n= 4-6. ^###^p<0.001 vs control (CTR) *p< 0.05 or **p<0.01 vs Aβ_1-42_ + anti-PGRN and ^Φ^p< 0.05 AZP2006 vs AZP2006 + anti-PGRN**.**

**
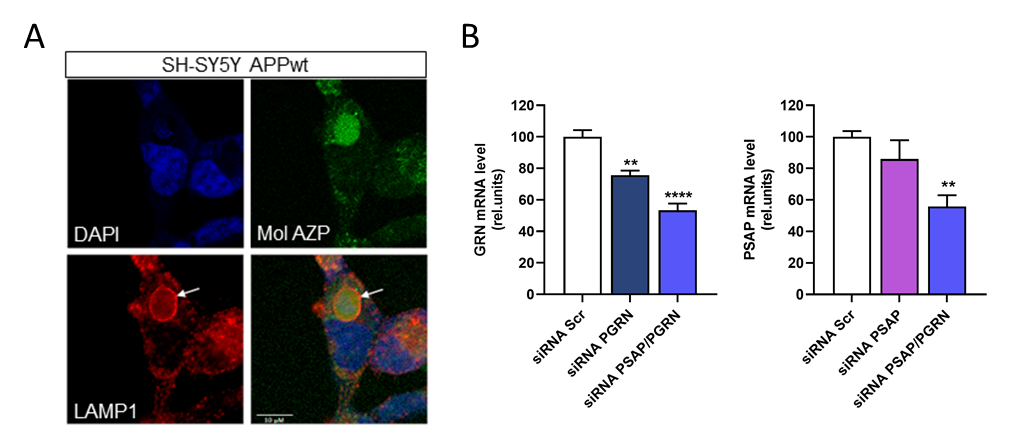
**

**Supplementary Figure 3: A**, AZP2006 (green) localized into lysosome vesicles (LAMP1 positive, red). **B**, **GRN and PSAP downregulation after specific siRNA transfection in rat primary cortical neurons**. On day 12 of culture, cells were transfected with the siRNA scramble (control mismatch) or with the siRNA targeting PGRN, PSAP or PGRN+PSAP at 20 nM (L-090442-02-0010, L-080037-02-0010, Dharmacon, Horizon Discovery Group plc, Cambridge Research Park, United Kingdom). The transfection was performed with the kit INTERFERin® (Polyplus transfection, Illkirch, France). Total cellular RNA was isolated using Kit Nucleospin RNA XS (Macherey-Nagel, Eupen, Belgium) according to the manufacturer’s instructions. Total RNA (100 ng) was transcribed into cDNA using SensiFAST™ cDNA Synthesis Kit (Bioline Meridian, Saint-Cyr-l’Ecole, France). The cDNAs were amplified with the following specific primers: GRN (forward, 5’-GAGTCGGACGCAGGCA-3’; reverse, 5’-ACAGGGCAGAACTGACC-3’), PSAP (forward, 5’-GCATGGCCGACATATGC-3’; reverse, 5’-AACCAGCGCACAGATCT-3’). The results were normalized to GAPDH (forward, 5’-ATCACCATCTTCCAGGA-3’; reverse, 5’-TGCATTGCTGACAATCTT-3’). All values are expressed as mean +/- SEM (standard error of the mean). N = 4. **p<0.01 and ****p<0.0001 vs siRNA scramble with One-way ANOVA followed by Fisher’s test.

**
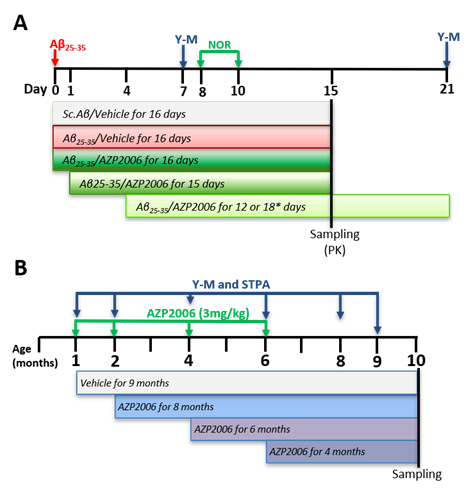
**

**Supplementary Figure 4:**

**Schematic representation of AZP2006 treatment timing. A,** AZP2006 treatment design in mice intoxicated with Aβ. **B**, AZP2006 treatment design in SAMP8 mice.

*
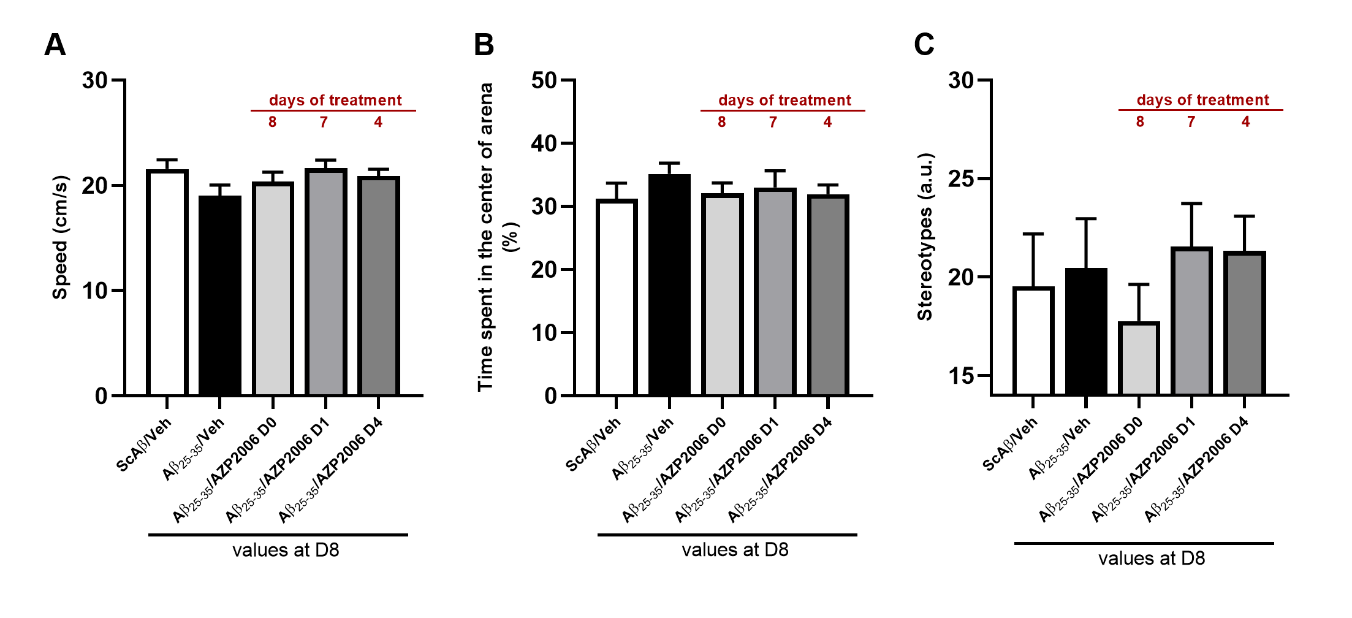
*

**Supplementary Figure 5: Locomotor behavior measured at the open field test.** Some locomotion parameters, such as speed, time spent in the center of the arena and stereotypic behavior were measured in C57B/6Rj mice after *icv* Aβ_25-35_ injection and/or treatment with AZP2006 from D0, D01 or D04. No differences were proved respect with control mice (Scramble Aβ).

**
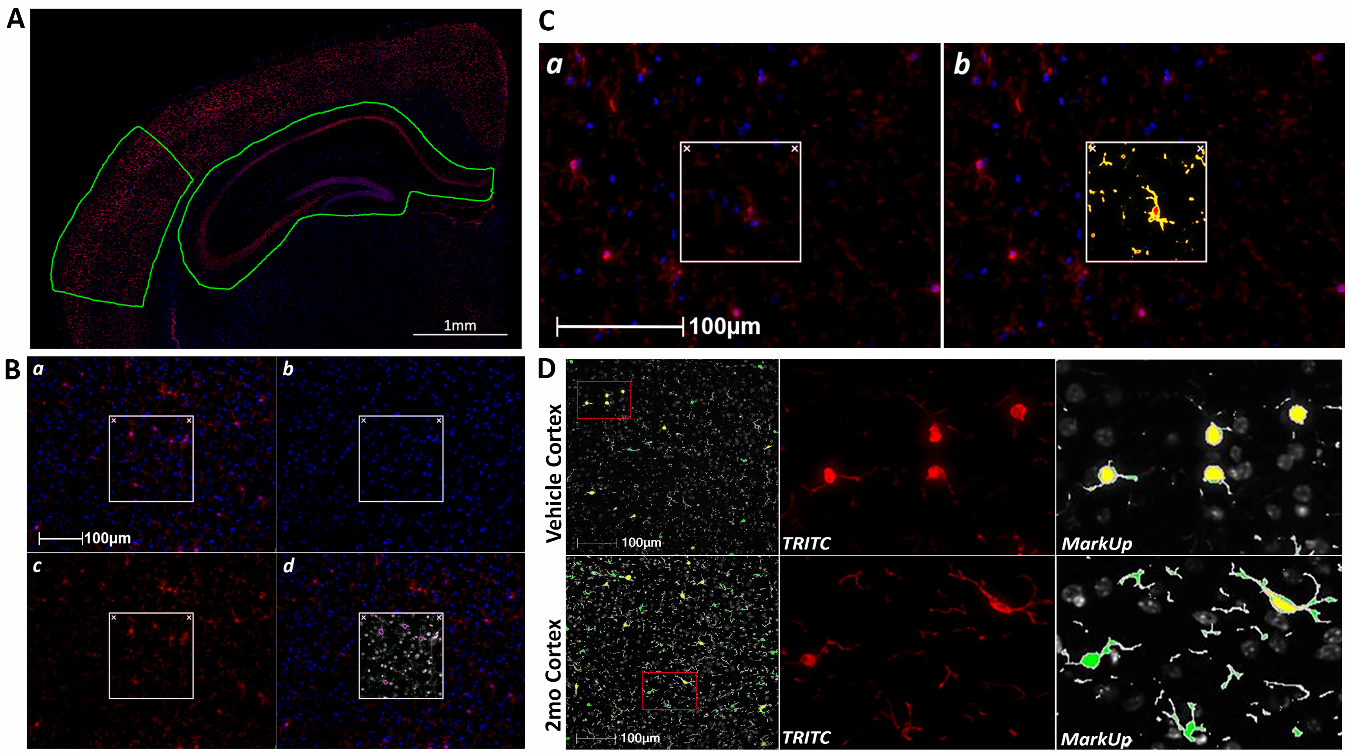
**

**Supplementary Figure 6: Microglia staining with Iba1 antibody. A**, Cortex and hippocampus regions (green line) considered for immunostaining quantification. **B, *a***: Iba-1 positive cells and DAPI; ***b***: DAPI alone; ***c***: Iba-1 immunostaining alone; ***d***: merge and segmentation. Only the overlapped staining was counted as microglia cell. Iba1 positive microglia were automatically counted with the Cytonuclear FL module of HALO software. **C**, the immunofluorescent positivity was measured in a radius around the nuclei of 1.5µm, in this way both cytoplasmic area and arborization were measured. In order to discriminate the cytoplasmic area from the microglia processes, two thresholds were considered: one able to detect weak staining (mostly microglia processes, in yellow, ***b***) and another for strong staining (microglia cell bodies, in red, ***b***). **D**, example of microglia size discrimination by Object Colocalization FL v1.0 module in the cortex of SAMP8 mice treated with the vehicle solution or AZP2006 at 3mg/kg/day during 8 months (treatment started at the age of 2 months). In yellow the activated microglia with a body size over a predetermined positivity threshold (Markup). In green inactivated microglia under the threshold value.

**
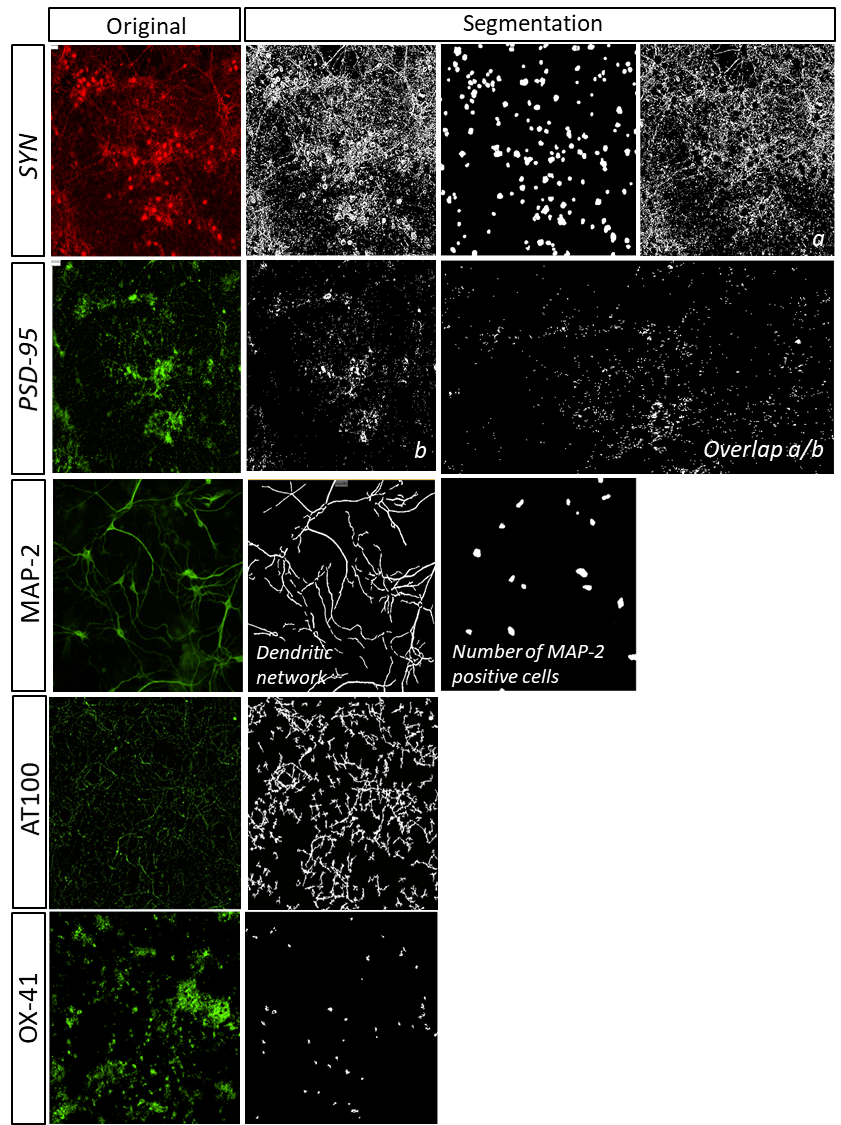
**

**Supplementary Figure 7: Staining and segmentation for fluorescence quantification by Custom Module Editor (Molecular Devices).** For each condition, between 30 and 40 pictures per well were taken using ImageXpress (Molecular Devices, San Jose, USA) with 20x magnification. All images were generated using the same acquisition parameters and analyses were directly and automatically performed by using Custom Module Editor (Molecular Devices) after segmentation (figure in black and white). The automated devises used in this study permitted to fully quantify spatial associations, especially when the patterns were indistinct, irregular, or highly punctate.
